# Hemodynamics during the 10-minute NASA Lean Test: evidence of circulatory decompensation in a subset of ME/CFS patients

**DOI:** 10.1186/s12967-020-02481-y

**Published:** 2020-08-15

**Authors:** Jihyun Lee, Suzanne D. Vernon, Patricia Jeys, Weam Ali, Andrea Campos, Derya Unutmaz, Brayden Yellman, Lucinda Bateman

**Affiliations:** 1 The Bateman Horne Center of Excellence, 24 South 1100 East, Suite 205, Salt Lake City, UT 84102 USA; 2 Jackson Laboratory for Genomic Medicine and University of Connecticut School of Medicine, Farmington, CT 06032 USA

**Keywords:** ME/CFS, Circulatory decompensation, Orthostatic intolerance, 10-minute NASA lean test, Point-of-care

## Abstract

**Background:**

Lightheadedness, fatigue, weakness, heart palpitations, cognitive dysfunction, muscle pain, and exercise intolerance are some of the symptoms of orthostatic intolerance (OI). There is substantial comorbidity of OI in ME/CFS (Myalgic Encephalomyelitis/Chronic Fatigue Syndrome). The 10-minute NASA Lean Test (NLT) is a simple, point-of-care method that can aid ME/CFS diagnosis and guide management and treatment of OI. The objective of this study was to understand the hemodynamic changes that occur in ME/CFS patients during the 10-minute NLT.

**Methods:**

A total of 150 ME/CFS patients and 75 age, gender and race matched healthy controls (HCs) were enrolled. We recruited 75 ME/CFS patients who had been sick for less than 4 years (< 4 ME/CFS) and 75 ME/CFS patients sick for more than 10 years (> 10 ME/CFS). The 10-minute NLT involves measurement of blood pressure and heart rate while resting supine and every minute for 10 min while standing with shoulder-blades on the wall for a relaxed stance. Spontaneously reported symptoms are recorded during the test. ANOVA and regression analysis were used to test for differences and relationships in hemodynamics, symptoms and upright activity between groups.

**Results:**

At least 5 min of the 10-minute NLT were required to detect hemodynamic changes. The < 4 ME/CFS group had significantly higher heart rate and abnormally narrowed pulse pressure compared to > 10 ME/CFS and HCs. The < 4 ME/CFS group experienced significantly more OI symptoms compared to > 10 ME/CFS and HCs. The circulatory decompensation observed in the < 4 ME/CFS group was not related to age or medication use.

**Conclusions:**

Circulatory decompensation characterized by increased heart rate and abnormally narrow pulse pressure was identified in a subgroup of ME/CFS patients who have been sick for < 4 years. This suggests inadequate ventricular filling from low venous pressure. The 10-minute NLT can be used to diagnose and treat the circulatory decompensation in this newly recognized subgroup of ME/CFS patients. The > 10 ME/CFS group had less pronounced hemodynamic changes during the NLT possibly from adaptation and compensation that occurs over time. The 10-minute NLT is a simple and clinically useful point-of-care method that can be used for early diagnosis of ME/CFS and help guide OI treatment.

## Background

Myalgic encephalomyelitis/chronic fatigue syndrome (ME/CFS) is a debilitating disease with significant unmet medical needs that affects as many as 2.5 million people in the U.S. and causes enormous burden for patients, their caregivers, the healthcare system and society. The symptoms of impaired function accompanied by severe fatigue, unrefreshing sleep, cognitive impairment and orthostatic intolerance are worsened by physical and cognitive exertion causing post-exertional malaise (PEM) [1]. ME/CFS is generally considered to be a post-viral or post-infection syndrome with immune, metabolic and neurologic sequelae [2]. Between 84 and 91% of ME/CFS patients are not yet diagnosed [3]. At least one-quarter of ME/CFS patients are house- or bedbound at some point in their lives [4]. The economic impact of ME/CFS is $17–$24 billion annually for direct costs and $9.1 billion from lost household and labor force productivity [5, 6].

Orthostatic intolerance (OI) is defined as the development of symptoms during upright posture that are relieved by lying down or reclining. Lightheadedness, headache, fatigue, weakness, heart palpitations, tremor and exercise intolerance are some symptoms of acute OI [7]. Chronic OI may present even more subtly with nausea, neurocognitive deficits, sensitivity to heat, or sleep problems [8]. Various physiological irregularities and syndromes can underlie orthostatic symptoms including postural orthostatic tachycardia syndrome (POTS), orthostatic hypotension (OH), neurally mediated hypotension and each may have subgroups of their own (i.e. post-viral POTS, hyperadrenergic POTS, neurogenic orthostatic hypotension, etc.) [9].

While questions still exist concerning the exact role of OI in ME/CFS, increasing evidence shows substantial comorbidity. Indeed, the Institute of Medicine (IOM) published clinical diagnostic criteria for ME/CFS listing OI as one of the core features of the illness [1]. Head-up tilt table testing, and continuous heart rate monitoring are used in research of OI syndromes. However, neither of these modalities is readily available to clinicians or easily performed in the clinic. Standardized methods for point-of-care diagnosis and testing for ME/CFS are needed.

Simple yet promising bedside tools to acquire orthostatic vital signs are standing or “leaning” tests (done with shoulders touching a wall for stability), wherein a patient’s heart rate (HR) and blood pressure (BP) are measured at set intervals before and after they move from a supine to standing position. Adoption of practical point-of-care tests for OI are important to improve ME/CFS diagnosis, identify possible causes of OI, and direct treatment options.

Our aim was to determine the utility of the 10-minute NLT as a point-of-care assessment of OI to aid in ME/CFS diagnosis and to gain an understanding of the hemodynamic changes associated with OI in ME/CFS. We found that the 10-minute NLT is easily administered in the clinic and is useful for diagnosing hemodynamic abnormalities that can be treated by physicians. Furthermore, use of the 10-minute NLT helped identify circulatory decompensation in a subgroup of ME/CFS patients. This is a newly recognized OI subgroup of ME/CFS patients that can be diagnosed with the 10-minute NLT and treated appropriately.

## Methods

### Participants

150 ME/CFS patients who had been seen at Bateman Horne Center (Salt Lake City, UT) for routine clinical care between February 2018 and September 2019 and 75 matched HCs were recruited for the study. The 150 MECFS subjects included 75 sick with ME/CFS for < 4 years (< 4 ME/CFS) and 75 sick for greater than 10 years (> 10 ME/CFS). The age range of ME/CFS participants and HCs was 18–65 years at the time of informed consent. HCs were matched with < 4 ME/CFS participants by age (± 5 years), gender and ethnicity. Enrolled ME/CFS participants were required to fulfill the International Chronic Fatigue Syndrome Study Group research criteria [10], the Canadian Consensus Criteria [11], and the IOM clinical diagnostic criteria [1]. HCs were recruited from the Salt Lake City metropolitan area using advertisements posted on social media, the clinic webpage or by phone contact with a volunteer pool from previous studies. HCs were considered generally healthy and between 18 and 65 years of age. HCs were excluded if they fulfilled ME/CFS diagnostic criteria or had a history of illness, had a BMI > 40 or had been treated with long-term (longer than 2 weeks) antiviral medication or immune modulatory medications within the past 6 months or had been treated with short-term (less than 2 weeks) antiviral or antibiotic medication within the past 30 days.

### Baseline (Year 1) assessments

All research participants had a physical examination at the baseline visit in Year 1 that included evaluation of vital signs, body mass index (BMI), orthostatic vital signs, skin, lymphatic system, HEENT, pulmonary, cardiac, abdomen, musculoskeletal, nervous system and fibromyalgia (FM) tender points. Medical history and concomitant medications were documented. The 10-minute NLT was conducted following the physical examination. The 10-minute NLT is an orthostatic challenge standing lean test. It requires an exam table, a pulse oximeter placed on one hand and a blood pressure cuff placed on the opposite arm. Objective hemodynamic parameters (i.e. HR and BP) were recorded while subjects were resting supine for 10 min, then moved to a standing position and remained standing for 10 min. Testing requires at least one person to scribe and monitor HR, signs, and symptoms and one person to obtain BP measurements. Participants were first asked to lie down undisturbed for 10 min. The participant was then asked to stand and lean against a wall with only shoulder blades touching and heels 6–8 inches from the wall. BP and HR are recorded every minute for 10 min and participants were asked to report any symptoms they experienced.

In addition, all subjects completed the following questionnaires before their baseline visit using REDCap, a secure and HIPAA compliant electronic data capture system; DePaul Symptom Questionnaire, Post-Exertional Fatigue Questionnaire, RAND-36, Fibromyalgia Impact Questionnaire-R, ACR 2010 Fibromyalgia Criteria Symptom Questionnaire, Pittsburgh Sleep Quality Index, Stanford Brief Activity Survey, Orthostatic Intolerance Daily Activity Scale, Orthostatic Intolerance Symptom Assessment, Brief Wellness Survey, Hours of Upright Activity, medical history and family history.

### Hemodynamic definitions

Systolic BP (SBP), diastolic BP (DBP) and HR were used as raw values recorded during the 10-minute NLT. Pulse pressure (PP) and narrowed pulse pressure (NPP) were calculated according to the consensus equation: PP = SBP- DBP and NPP = PP/SBP. PP is considered abnormally narrow if it is less than 25% of SBP [12]. Subgroup analysis of < 4 and > 10 ME/CFS participants was conducted. For assessing OI manifestations, changes in HR and BP during the 10-minute NLT were classified as follows. OH was defined as a decrease in SBP of 20 mm Hg or more, or a decrease in DBP of 10 mm Hg or more in the first 3 min standing compared to resting supine values. POTS was defined as an increase of HR greater than 30 bpm or a HR of greater than 120 bpm, based on the average of the last three minutes standing. (e.g., if the standing portion of the test was terminated at 6 min, we calculated the mean of the data recorded during the 3rd, 4th and 5th minutes). This allowed us to include results from participants unable to complete the 10-minute NLT. OI symptoms were spontaneously reported by participants during the test. OI signs were observed and recorded by the scribe. We conceptualized OI symptoms by identifying and refining common words or concepts mentioned by participants. To assess the relationship between self-reported hours of upright activity (HUA) and hemodynamic changes during the 10-minute NLT, multiple linear regression was performed between the average values of hemodynamics data: SBP, DBP, HR, PP, and NPP in last 3 min standing. Also, additional multiple logistic regression was performed for comparing hemodynamics between ME/CFS patients with < 5 HUA and > 5 HUA. HUA is defined as time spent with feet on the floor (sitting, standing, walking) in the last 24 h.

### Statistical analysis

Data were analyzed by Excel and STATA/MP 13.1 Windows [32-bit] version. All demographic data of subgroups were compared using ANOVA or T-test, where appropriate. Repeated measures of ANOVA were used to detect differences in hemodynamic data of each subgroup in each minute and presented as Means (SD). Fisher’s Exact test was applied to demonstrate association between comprehensive hemodynamics trend results in each subgroup. Descriptive statistics used for spontaneous OI symptom reports. The relationship between the hemodynamic response and HUA was modeled by multiple linear regression after adjusting for age and gender. Effect modification by medication was evaluated via stratified analyses and tested via the interaction term approach. Logistic regression was used to analyze HUA. T-test, F-test and Chi-Square test were used for validating models. A p-value of < 0.05 was considered statistically significant.

## Results

Table 1 shows the baseline demographics and clinical characteristics of the study participants. The < 4 ME/CFS participants and HCs were similar in age and > 10 ME/CFS participants were older (P < 0.005). The distribution of race and gender between the 3 groups was similar. The average HUA was 6.05 (SD ± 4.02) for all ME/CFS participants. There was a significant difference in HUA between < 4 ME/CFS and > 10 ME/CFS participants (5.36 SD ± 3.65 compared to 6.75 SD ± 4.28, P < 0.05). The baseline HR was significantly different between all ME/CFS participants and HCs (P < 0.0001) and between < 4 ME/CFS and > 10 ME/CFS (P < 0.05). There was no difference in baseline supine SBP and DBP between groups.

**Table 1:** Participant characteristics

	HCs (n = 75)	ME/CFS (n = 150)	< 4 ME/CFS (n = 75)	> 10 ME/CFS (n = 75)
Age (SD)	38.5 (± 14)^c^	42.1 (± 13)^c^	39.0 (± 13)^b^	45.3 (± 12)^b^
Race % (n)				
White	97 (73)	98 (147)	99 (74)	97 (73)
Other	3 (2)	2 (3)	1 (1)	3 (2)
Gender % (n)				
Male	28 (30)	40 (42)	31 (23)	25 (19)
Female	72 (45)	60 (108)	69 (52)	75 (56)
HUA (SD)	N/A	6.05 (± 4)	5.36 (± 4)^c^	6.75 (± 4)^c^
Supine vitals (SD)				
SBP	116.45 (± 15)	116.67 (± 15)	116.21 (± 14)	117.15 (± 15)
DBP	75.54 (± 10)	76.73 (± 10)	76.89 (± 9)	76.57 (± 11)
HR	62.64 (± 11)^a^	71.25 (± 12)^a^	73.55 (± 11)^c^	68.9 (± 12)^c^

^a^P-Value < 0.0005

^b^P-Value < 0.005

^c^P-Value < 0.05

**Fig. 1:**
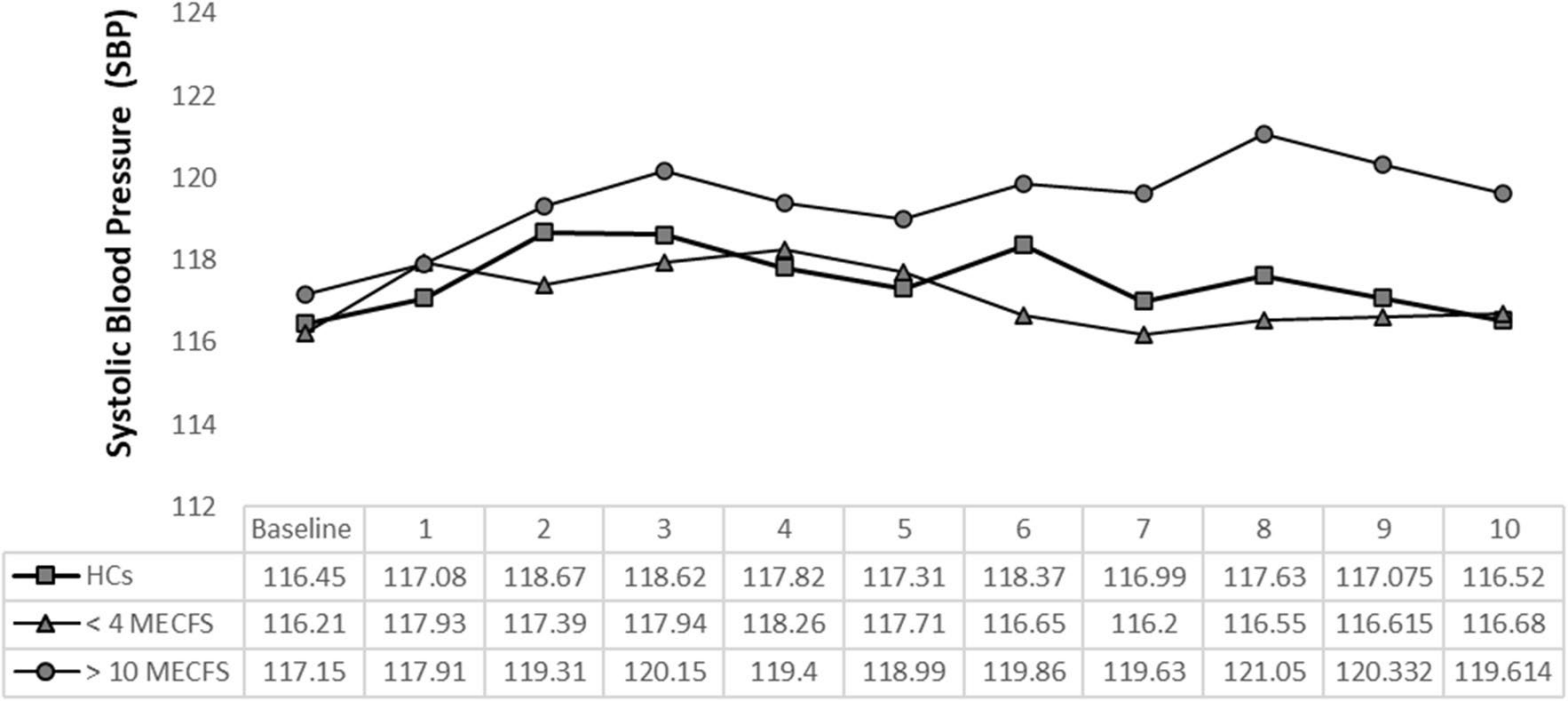
Systolic blood pressure (SBP) changes during the 10-minute NLT. Baseline blood pressure was measured while participants rested supine, then every minute upon standing for the 10-minute NLT. SBP was not significantly different between the three groups. However, the SBP for > 10 ME/CFS patients was higher than < 4 ME/CFS and HCs and remained high for the duration of the 10-minute NLT

**Fig. 2:**
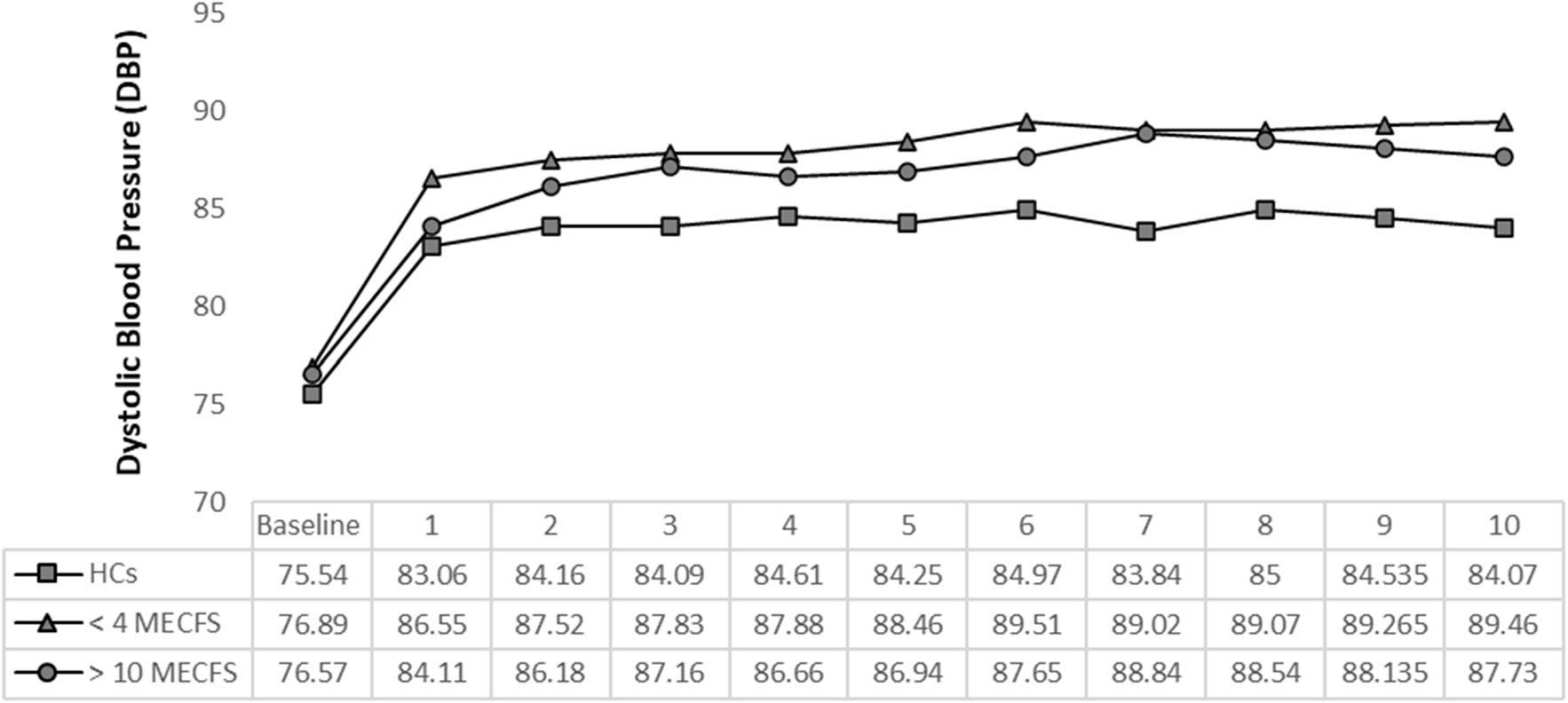
Diastolic blood pressure (DBP) changes during the 10-minute NLT. Baseline blood pressure was measured while participants rested supine, then every minute upon standing for the 10-minute NLT. All participants had a similar, sudden increase in DBP immediately after standing. (Baseline DBP: < 4 ME/CFS 76.89 SD ± 8.89, > 10 ME/CFS 76.57 SD ± 10.56, HCs 75.54 SD ± 10.18; DBP after standing 1 min: < 4 ME/CFS 86.55 SD ± 10.69, > 10 ME/CFS 84.11 SD ± 8.74, HCs 83.06 SD ± 10.38). DBP increased significantly for < 4 and > 10 ME/CFS after 3 min standing compared to HCs

**Fig. 3:**
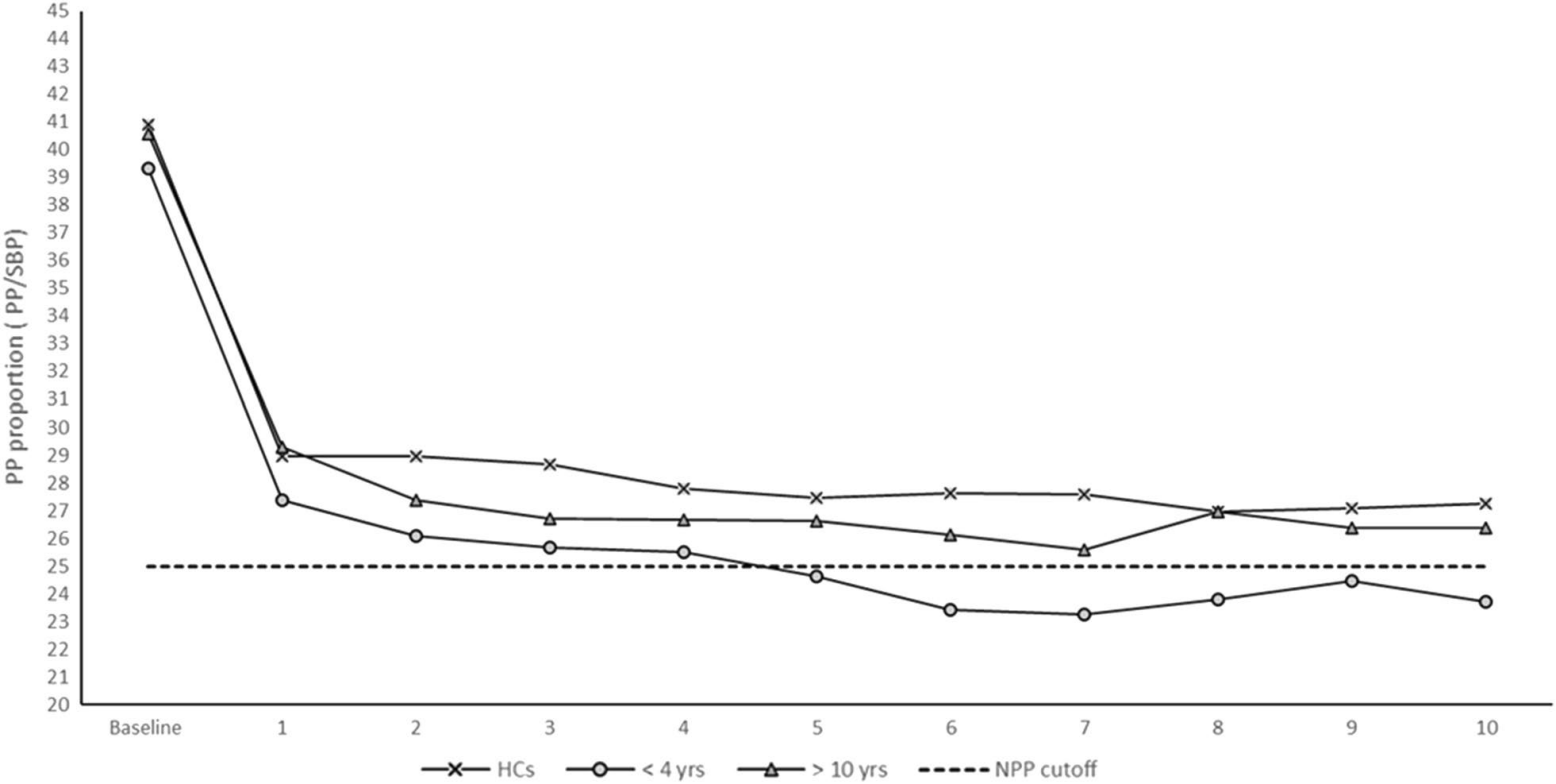
Change in pulse pressure during the 10-NLT. All participants had a sudden decrease in PP in the first minute after transitioning from lying down to standing. Abnormal NPP, defined as PP/SBP of < 25%, occurred in < 4 ME/CFS patients after 5 min (< 4 ME/CFS 24.63% SD ± 7.71, > 10 ME/CFS 26.63% SD ± 7.85, HCs 27.47% SD ± 7.94, P > 0.11) and was significantly different from > 10 ME/CFS and HCs 6 min into the 10-minute NLT

**Fig. 4:**
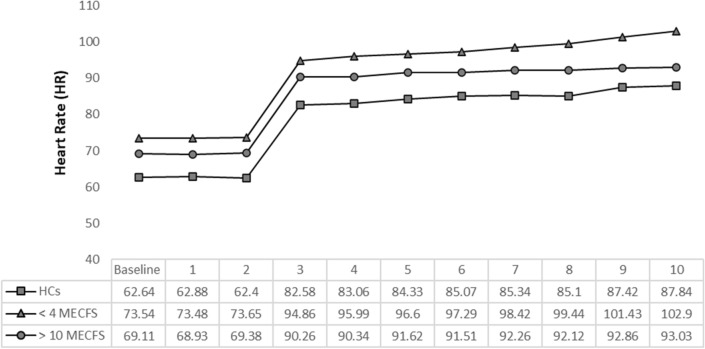
Heart rate changes during the 10-minute NLT. Patients with ME/CFS demonstrated significant HR increases every minute compared to HCs even though the three groups showed continuous HR increases throughout the 10-minute NLT. Baseline HR and HR throughout the test was significantly higher for the < 4 ME/CFS group compared to > 10 ME/CFS and HCs

We used the 10-minute NLT to evaluate hemodynamics in all 225 participants. The > 10 ME/CFS group showed a higher, increased trend of SBP compared to < 4 ME/CFS and HCs but the difference was not statistically significant (P > 0.11, Fig. 1). All participants had a similar, sudden increase in DBP immediately after standing. The < 4 and > 10 ME/CFS had significantly greater increases in DBP after 3 min compared to HCs (P > 0.05, Fig. 2). All participants had a sudden decrease in PP in the first minute of standing but there were no significant differences between groups. Abnormal NPP (PP/SBP < 25%) occurred in the < 4 ME/CFS group after 5 min (< 4 ME/CFS 24.63% SD ± 7.71, > 10 ME/CFS 26.63% SD ± 7.85, HCs 27.47% SD ± 7.94, P > 0.11) and by 6 min the < 4 ME/CFS have statistically significant NPP compared to the other groups (F_2,186_ = 5.33, P > 0.006) (Fig. 3). Patients with ME/CFS demonstrated significant HR increases every minute compared to HCs even though all subgroups showed continuous HR increases as time standing progressed (Fig. 4). The < 4 ME/CFS group had higher baseline HR compared to > 10 ME/CFS and HCs (< 4 ME/CFS 73.55 SD ± 11.24, > 10 ME/CFS 68.9 SD ± 12.15, HCs 62.64 SD ± 10.89, F_2,220_ = 17.18, P > 0.00001) and significantly higher HR compared to > 10 ME/CFS throughout the 10-minute NLT (P < 0.05). Spontaneous symptoms of muscle weakness, tingling, dizziness/lightheadedness and headache were more frequently reported by ME/CFS patients while HCs reported headache, dizziness, light headedness, or nausea. Only one HC reported muscle weakness or tingling. Interestingly, presyncope and syncope occurred in 12% of HCs and 8% of both < 4 and > 10 ME/CFS participants.

The 10-minute NLT induced OI manifestations of POTS and OH in 54% of ME/CFS participants and 49% of HCs (Table 2). Further, HR increased in all groups for each minute of standing so that by 10 min 33% of HCs, 42% < 4 MECFS and 38% > 10 MECFS met POTS criteria. More ME/CFS participants spontaneously reported symptoms compared to HCs (P < 0.05). Even though the rate of POTS was similar among ME/CFS and HCs, significantly fewer HCs reported OI symptoms (P < 0.005). Spontaneous OI symptom report in those without POTS or OH was greater in ME/CFS participants compared to HCs (P < 0.0005).

**Table 2:** OI manifestations during the 10-minute NLT

	HCs (n = 75) (%)	ME/CFS (n = 150) (%)	< 4 ME/CFS (n = 75) (%)		> 10 ME/CFS (n = 75) (%)
Hemodynamics					
POTS or OH any time sequence	49	54	60	48	
POTS or OH last 3 min standing	35	40	47	33	
POTS					
* 1 min standing*	15	15	12	19	
* 3 min standing*	19	25	27	23	
* 5 min standing*	23	28	31	26	
* 10 min standing*	33	40	42	38	
OH					
* SBP decrease of 20 mm Hg or more*	1	6	2	1	
* DBP decrease of 10 mm Hg or more*	0	1	1	0	
OI symptoms					
OI symptoms reported	21^a^	55^a^	63^c^	48^c^	
OI symptoms PLUS signs	8^b^	25^b^	31	20	
OI signs ONLY	31	27	28	25	
OI symptoms ONLY	20^a^	50^a^	60	42	
POTS with symptoms	33	36	40	32	
OH with symptoms	1	3	5	1	

^a^P-Value < 0.0005

^b^P-Value < 0.005

^c^P-Value < 0.05

We determined whether hemodynamic changes during the 10-minute NLT were due to medication. Similar results to those shown in Figs. 2 and 4 were found. ME/CFS participants not taking medications had a significantly higher baseline HR compared to HCs (P < 0.001). ME/CFS not taking prescription medications for OI had significantly different DBP (P < 0.009), HR (P < 0.001) and NPP (P < 0.04) during the last 3 min standing compared to HCs.

Logistic regression was used to determine whether HR was predictive of upright activity. A HR increase of 1 beat per minute resulted in 0.06 HUA decrease (P = 0.002). Therefore, we would predict HUA would decrease by 1.8 h with a HR increase of 30 beats per minute. Indeed, HUA decreased 1.4 h in ME/CFS participants with POTS (P > 0.05, F = 0.17). HUA decreased 2.14 h in those ME/CFS participants with POTS or OH plus spontaneously reported symptoms (P = 0.005, F = 0.03). However, having POTS did not predict decreased HUA (P > 0.13, χ^2^ = 0.13).

## Discussion

Our study demonstrated that the 10-minute NLT is a simple and meaningful point-of-care test that can be administered in a clinic setting by trained medical assistants or nurses supervised by a clinician. We also showed that it takes at least 5 min of standing/leaning to detect meaningful hemodynamic changes and the full 10 min are likely to detect more. This is important because physicians generally do 1–3 min of standing in a routine orthostatic assessment. Van Campen et al. have shown that abbreviated Tilt Table testing of only 2–5 min misses a substantial proportion of those ultimately diagnosed with POTS during a 10-min Tilt Table test [13]. Plash et al. compared Tilt Test versus Standing hemodynamics in POTS and state that while Tilt Testing produced larger increases in heart rate, both Tilt and Stand tests done for 10 min provide meaningful data [14]. Sensitivity of the 30 bpm criterion was similar for both tests (TILT-10 = 93%, STAND10 = 87%), but Specificity was less at 10 min for TILT (40%) than 10 min STAND (67%). They caution that a diagnosis of POTS should include other OI criteria and not be based solely on increases in heart rate regardless of test used. It is important to emphasize that Tilt Table testing is not readily available to all ME/CFS patients, and protocols and interpretation of results differ from center to center. That is why an in-office 10-minute NLT is important. We propose that the objective measurements obtained from the 10-minute NLT are clinically useful in symptomatic patients regardless of the cause and provide direction for supportive care. While it is likely that chronic OI in ME/CFS is compounded by severe deconditioning, this cannot be the only cause, as many experience OI from the early onset of disease, even if fit and active at the time they become ill.

The high occurrence of syncope and increased heart rate of > 30 bpm in HCs during the 10-minute NLT was not anticipated but is not surprising. The 10-minute NLT very effectively removes the “muscle pump” leaving only the neurovascular response to the completely vertical orthostatic stress (that is leaning for 10 min). It is noteworthy that many “healthy” people experience syncope during head up tilt table testing [15] and tilt table tests are performed at a 70% angle whereas the 10-minute NLT is totally vertical. A HR increase of > 30 during the 10-minute NLT was the most common orthostatic change in vital signs for all subjects. One explanation might be that all study participants were required to arrive fasting for a blood draw done about 30 min before the 10-minute NLT and might explain increased rates across all groups (fasting glucose levels were similar among groups, data not shown). It is important to emphasize, however, that spontaneous reporting of OI symptoms during the 10-minute NLT was much more common and diverse in the ME/CFS patients than HC.

OI is a common chronic complaint in the ME/CFS population that only occasionally occurs in otherwise healthy people (usually an acute problem triggered by heat, dehydration or emotional triggers that are easily remedied). One clear indicator of the chronic pervasive nature of OI in ME/CFS is self-reported HUA. Although HUA data were not collected in healthy controls, one can estimate at least 12–15 HUA on average in healthy individuals. In this study the average HUA for all ME/CFS participants was 6 h per 24-h period. The combination of OI symptoms induced by upright posture with low HUA in ME/CFS strongly support an underlying chronic disorder of OI. In addition, our study also demonstrates that the underpinnings of OI in ME/CFS are more complex than just meeting criteria for POTS.

Higher heart rates (both resting and standing) and a pathologic drop in pulse pressure stand out as distinguishing markers of illness between the ME/CFS and HCs. Almost every measured response (other than syncope or presyncope) to orthostatic stress was most dramatic in the < 4 ME/CFS, followed by > 10 ME/CFS and the least in HC. Blood pressure and heart rate variability have been proposed as potential biomarkers of ME/CFS. Frith et al. [16] used tilt table testing and identified blood pressure variability in ME/CFS and proposed it as a bedside diagnostic tool to measure abnormal hemodynamics. A study that used a 20-min standing test identified heart rate variability as a potential marker of fatigue in ME/CFS with POTS [17]. The ME/CFS POTS patients in this study had a shorter duration of illness (average 6 years) compared to the ME/CFS only group (average 10 years). Finally, Richardson et al. [18] used the same 20-min standing test but without hemodynamic assessment and detected an increase in Activin B in ME/CFS but not in healthy controls in response to the orthostatic challenge that correlated with ME/CFS disease severity. This pattern suggests that those with longstanding ME/CFS illness have adapted to their condition in some ways.

The striking finding of abnormally narrowed pulse pressure in the < 4 ME/CFS group suggests a significant drop in ventricular stroke volume while standing in place that can only be explained by a relative hypovolemia. Typical causes of an abnormally narrowed pulse pressure are constrictive (cardiac tamponade or restrictive pericarditis), outflow obstruction (aortic or pulmonary stenosis), pump failure from a diseased heart, or shock (anaphylactic, septic, cardiogenic) [12] and none of these are present in the ME/CFS patients enrolled in this study. The pressing research question raised by this study is what is causing the problem. Small studies that have not yet been replicated suggest autoantibodies against adrenergic and muscarinic receptors [19], or a neuroinflammatory process [20] may be playing a role. Invasive cardiopulmonary exercise test (iCPET) was used to show that ME/CFS patients have reduced atrial filling pressures and impaired systemic oxygen extraction during exercise compared to healthy people. This may be related to abnormal delivery of oxygen due to microscopic left to right shunt at the tissue level, and/or abnormal cellular uptake of oxygen [21]. We hypothesize that patients with ME/CFS have lower tolerance of the perfusion changes induced by orthostatic stress, from autonomic nervous system dysregulation in combination with abnormal cellular energy metabolism.

While it isn’t entirely clear why the > 10 ME/CFS group appear to tolerate the orthostatic stress better than < 4 ME/CFS in terms of a dramatic drop in pulse pressure, that does not prove they aren’t experiencing a drop in cerebral perfusion. It is possible that after many years of ME/CFS illness there is gradual adaptation of the circulatory stress response to upright posture. That may also explain why the > 10 ME/CFS had the highest rise in SBP during the 10-minute NLT. There is also an age difference between the < 4 and > 10 ME/CFS groups of about 5–6 years which might also explain the higher SBP response in the > 10 ME/CFS subgroup. A very important recently published study demonstrated that cerebral blood flow is reduced in ME/CFS during head-up tilt testing even in the absence of hypotension or tachycardia [22]. This is consistent with our findings and may explain why ME/CFS patients were not much more likely to meet standard criteria for POTS or OH than the HCs, even though they still became more symptomatic during the 10-minute NLT.

This study has limitations. The study originally aimed to recruit 150 ME/CFS patients sick for < 4 years and was amended after 1 year to recruit 75 < 4 and 75 > 10 ME/CFS for comparison. By this time the HCs had been recruited and matched with the < 4 year ME/CFS. This is why the > 10 ME/CFS group is older and not matched with HCs. Participants were not required to taper off all medications in advance of the 10-minute NLT, although many did not take their morning medications due to fasting for the blood draw. This should potentially have blunted the response to the 10-minute NLT making the problem look less severe, or conversely, by skipping morning meds and fasting, may have caused rebound symptoms making the results look worse. It is unclear why ME/CFS on OI medications had more severe OI findings, but it’s possible that more severe underlying OI led to the use of these medications as well as a more dramatic response to withholding meds for the fasting blood draw.

## Conclusion

In conclusion, the use of the point-of-care 10-minute NLT identified circulatory decompensation in a subgroup of ME/CFS patients sick for < 4 years. The 10-minute NLT is a simple, in-office assessment that can be used for early diagnosis and management of ME/CFS. The most important take-home clinical message from this study is that the identification of OI in ME/CFS will immediately provide information for objectively supported diagnoses and evidence-based targeted treatment, drawing from a large literature regarding OI and POTS, even if the cause is unknown.

## Data Availability

The datasets used and/or analyzed are available from the corresponding author upon request.

## Abbreviations

OI: Orthostatic intolerance
ME/CFS: Myalgic Encephalomyelitis/Chronic Fatigue Syndrome
NLT: NASA Lean Test
HCs: Healthy controls
POTS: Postural orthostatic tachycardia syndrome
OH: Orthostatic hypotension
IOM: Institute of Medicine
HR: Heart rate
BP: Blood pressure
FM: Fibromyalgia
SBP: Systolic blood pressure
DBP: Diastolic blood pressure
PP: Pulse pressure
NPP: Narrowed pulse pressure
HUA: Hours of upright activity
